# Changes in Health Indicators Among Caregivers — United States, 2015–2016 to 2021–2022

**DOI:** 10.15585/mmwr.mm7334a2

**Published:** 2024-08-29

**Authors:** Greta Kilmer, John D. Omura, Erin D. Bouldin, Jenny Walker, Katie Spears, Janelle Gore, Akilah R. Ali, Lisa C. McGuire

**Affiliations:** ^1^Division of Population Health, National Center for Chronic Disease Prevention and Health Promotion, CDC; ^2^Division of Epidemiology, Department of Internal Medicine, University of Utah, Salt Lake City, Utah; ^3^Oak Ridge Institute for Science and Education, Oak Ridge, Tennessee; ^4^Nell Hodgson Woodruff School of Nursing, Emory University, Atlanta, Georgia.

**Summary**

**What is already known about this topic?**

One in five U.S. adults are caregivers to family members or friends with a chronic health condition or disability. Negative associations between caregiving and caregiver health are known.

**What is added by this report?**

Among caregivers, prevalence of four health indicators improved and six worsened from 2015–2016 to 2021–2022. Changes among caregivers were often similar to changes among noncaregivers, and most health indicators remained worse for caregivers. During 2021–2022, measures for 13 of 19 indicators were worse for caregivers than for noncaregivers.

**What are the implications for public health practice?**

Strategies for supporting caregivers are available. Integrating these strategies with existing programs to address mental health and chronic diseases among this population might improve caregiver well-being.

## Abstract

Caregivers provide support to persons who might otherwise require placement in long-term care facilities. Approximately one in five U.S. adults provides care to family members or friends who have a chronic health condition or disability. Promoting the well-being of this large segment of the population is a public health priority as recognized by the 2022 National Strategy to Support Family Caregivers. Although negative associations between caregiving and caregiver health are known, changes in the health status of caregivers over time are not. Data from the 2015–2016 and 2021–2022 Behavioral Risk Factor Surveillance System were analyzed to compare changes in the prevalence of 19 health indicators among cross-sectional samples of caregivers and noncaregivers at different time points. Caregivers experienced improvements in prevalence of four health indicators, whereas six worsened. Some health indicators, such as cigarette smoking, improved for both caregivers and noncaregivers, although smoking prevalence remained higher for caregivers (16.6% versus 11.7%). Prevalence of lifetime depression increased for both groups and remained higher among caregivers (25.6%) than among noncaregivers (18.6%). During 2021–2022, age-adjusted estimates for caregivers were unfavorable for 13 of the 19 health indicators when compared with noncaregivers. Strategies for supporting caregivers are available, and integrating these with existing programs to address mental health and chronic diseases among this population might improve caregiver well-being. For example, many community organizations support caregivers by offering interventions designed to relieve caregiver strain, including skills training, support groups, and care coordination.

## Introduction

Caregivers provide support to persons who might otherwise require placement in long-term care facilities. Approximately one in five U.S. adults provides regular care or assistance to a friend or family member with a health condition or disability (*1*). Promoting the long-term well-being of this large segment of the population is a public health priority as recognized by the first National Strategy to Support Family Caregivers (*2*). The time commitment and responsibilities of caregiving can place an undue emotional, economic, and physical burden on caregivers (*2*). During 2015–2017, caregivers in the generation born during 1946–1964 had more chronic health conditions and more frequent mental distress than noncaregivers of the same age (*3*). Although studies have described differences in health indicators between caregivers and noncaregivers (*4*,*5*), this report compares changes in the prevalence of 19 health indicators among caregivers and noncaregivers from 2015–2016 to 2021–2022.

## Methods

### Data Source

The Behavioral Risk Factor Surveillance System (BRFSS) is an annual, state-based, random-digit–dialed telephone survey of the noninstitutionalized U.S. adult population aged ≥18 years in all 50 states, the District of Columbia, and U.S. territories.* In addition to core questions administered to all participants, states can include optional modules. Data from the core BRFSS questionnaire and the optional caregiver module during 2015–2016 and 2021–2022 were assessed for 35 states and Puerto Rico, where the optional caregiver module was included at least once in both periods.^†^ This activity was reviewed by CDC, deemed not research, and was conducted consistent with applicable federal law and CDC policy.^§^ Among all respondents, 92,461 who responded “yes” to the question, “During the past 30 days, did you provide regular care or assistance to a friend or family member who has a health problem or a disability?” were classified as caregivers. Those who responded “no” or indicated that their care recipient died during the previous 30 days (353,242) were classified as noncaregivers; 2,489 who responded “did not know/not sure” or refused to answer were excluded. Estimates for both periods were available for demographic characteristics (sex, age, race and ethnicity, education level, employment status, marital status, home ownership, and annual household income) and the following 19 health indicators: 1) current cigarette smoking, 2) binge drinking,^¶^ 3) heavy drinking,** 4) physical inactivity,^††^ 5) fair or poor self-rated health, 6) frequent mental distress,^§§^ 7) frequent physical distress,^¶¶^ 8) lifetime diagnosed depression,*** 9) coronary heart disease, 10) stroke, 11) chronic obstructive pulmonary disease, 12) arthritis, 13) diabetes, 14) obesity, 15) diagnosed asthma, 16) any chronic physical health condition,^†††^ 17) multiple chronic physical health conditions, 18) having no health coverage,^§§§^ and 19) inability to see a doctor because of cost.^¶¶¶^

### Statistical Methods

Percentage point changes (changes) in the unadjusted prevalence of demographic characteristics and caregiving status by state during 2015–2016 and 2021–2022 were compared using *t*-tests. Logistic regression, with age-adjustment using a continuous age variable, was used to measure percentage point differences in health indicators (dependent variable) between caregivers and noncaregivers (independent variable) as well as changes in health indicators (with time indicator as independent variable). Logistic regression was used to determine whether age-adjusted changes among caregivers and noncaregivers were different (i.e., time interaction). Statistical tests with p-values <0.05 were considered significant. Analyses were conducted using SAS software (version 9.4; SAS Institute) and SAS-callable SUDAAN software (version 11.0.1; RTI International) to account for complex sample design and weighting.

## Results

The percentage of U.S. adults who were caregivers during 2015–2016 (20.2%) and 2021–2022 (20.1%) was similar; the percentage increased in three states and decreased in 11 states and Puerto Rico (Figure). The proportion of caregivers aged ≥60 years increased from 28.0% during 2015–2016 to 35.4% during 2021–2022. This increase was larger than among noncaregivers (28.8% to 31.5%) (Table 1).

**FIGURE:**
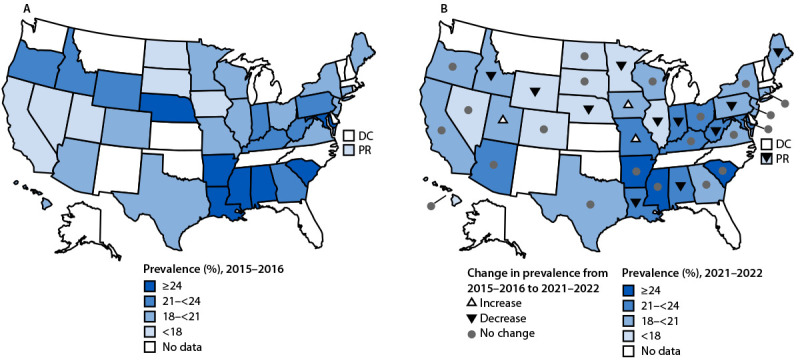
Prevalence of caregiving* (A) and changes in caregiving**^†^** (B) among U.S. adults — Behavioral Risk Factor Surveillance System, 35 states**^§^** and Puerto Rico, 2015–2016 to 2021–2022 **Abbreviations:** DC = District of Columbia; PR = Puerto Rico. * Provided regular care or assistance during the previous 30 days to a friend or family member who had a health problem or disability. ^†^ Prevalence for 2015–2016 compared with 2021–2022 using *t*-test. ^§^ Alabama, Arizona, Arkansas, California, Colorado, Connecticut, Georgia, Hawaii, Idaho, Illinois, Indiana, Iowa, Kentucky, Louisiana, Maine, Maryland, Minnesota, Mississippi, Missouri, Nebraska, Nevada, New Jersey, New York, North Dakota, Ohio, Oregon, Pennsylvania, South Carolina, South Dakota, Texas, Utah, Virginia, West Virginia, Wisconsin, and Wyoming.

**TABLE 1:** Changes in demographic characteristics among caregivers* and noncaregivers — Behavioral Risk Factor Surveillance System, 35 states^†^ and Puerto Rico, 2015–2016 and 2021–2022

Characteristic	Caregivers n = 92,461			Noncaregivers n = 353,242			Difference in change (caregivers minus noncaregivers)	
	2015–2016 (%)^§^	2021–2022 (%)^§^	Percentage point change	2015–2016 (%)^§^	2021–2022 (%)^§^	Percentage point change	Percentage point	p-value^¶^
**Sex**								
Female	57.9	59.2	1.3	49.9	49.8	−0.1	1.4	0.17
Male	42.1	40.8	−1.3	50.1	50.2	0.1	−1.4	0.17
**Age group, yrs**								
18–29	18.0	13.3	−4.7**	20.8	19.9	−0.9**	−3.8	<0.001
30–39	14.4	14.5	0.1	17.0	17.3	0.3	−0.2	0.81
40–49	17.8	15.9	−1.9**	16.2	15.6	−0.6	−1.3	0.14
50–59	21.9	21.0	−0.9	17.2	15.6	−1.6**	0.7	0.20
60–69	16.9	19.8	2.9**	14.9	15.3	0.4	2.5	<0.001
70–79	8.3	11.3	3.0**	9.2	10.9	1.7**	1.3	0.01
≥80	2.9	4.3	1.4**	4.8	5.3	0.5**	0.9	<0.001
**Race and ethnicity^††^**								
American Indian or Alaska Native	1.2	0.9	−0.3	0.8	0.8	0	−0.3	0.29
Asian	2.3	4.2	1.9**	5.7	6.9	1.2**	0.7	0.02
Black or African American	12.8	12.4	−0.4	11.0	10.9	−0.1	−0.3	0.70
Native Hawaiian or Pacific Islander	0.1	0.2	0.1	0.2	0.2	0	0.1	0.44
White	66.8	65.4	−1.4	61.5	58.8	−2.7**	1.3	0.27
Hispanic or Latino	14.3	14.1	−0.2	18.9	20.4	1.5**	−1.7	0.15
Other races	0.5	0.6	0.1	0.4	0.4	0	0.1	0.63
Multiple races	2.0	2.2	0.2	1.5	1.8	0.3**	−0.1	0.56
**Education level**								
Less than high school	11.4	8.7	−2.7**	15.0	12.9	−2.1**	−0.6	0.08
High school graduate	27.3	26.4	−0.9	28.1	27.2	−0.9**	0	0.98
Some college or technical school	36.1	34.8	−1.3	30.0	29.4	−0.6	−0.7	0.55
College graduate	25.2	30.1	4.9**	27.0	30.5	3.5**	1.4	0.10
**Employment status**								
Employed for wages	46.6	44.1	−2.5**	48.5	48.3	−0.2	−2.3	0.03
Self-employed	9.5	9.6	0.1	8.5	8.6	0.1	0.0	0.84
Out of work	7.0	8.0	1.0	5.3	6.1	0.8**	0.2	0.95
Homemaker	7.2	5.8	−1.4**	6.8	5.1	−1.7**	0.3	0.47
Student	4.4	3.4	−1.0**	5.8	5.3	−0.5	−0.5	0.18
Retired	17.8	22.3	4.5**	18.4	20.6	2.2**	2.3	0.001
Unable to work	7.7	6.8	−0.9**	6.7	5.9	−0.8**	−0.1	0.97
**Marital status**								
Married	52.0	55.7	3.7**	51.1	49.7	−1.4**	5.1	<0.001
Divorced or separated	14.5	13.5	−1.0	13.0	12.4	−0.6**	−0.4	0.58
Widowed	4.9	5.7	0.8**	7.3	7.5	0.2	0.6	0.10
Never married	22.9	20.3	−2.6**	24.0	25.1	1.1**	−3.7	<0.001
Unmarried couple	5.6	4.8	−0.8	4.6	5.3	0.7**	−1.5	0.004
**Own or rent home**								
Own	70.8	73.6	2.8**	67.0	67.6	0.6	2.2	0.02
Rent	23.2	21.2	−2.0**	27.0	26.2	−0.8	−1.2	0.14
Other arrangement	5.9	5.2	−0.7	6.0	6.2	0.2	−0.9	0.06
**Annual household income**								
<$10,000	4.9	3.0	−1.9**	5.2	3.5	−1.7**	−0.2	0.39
$10,000–$19,999	12.1	6.7	−5.4**	11.1	6.9	−4.2**	−1.2	0.10
$20,000–$34,999	17.1	16.2	−0.9	16.4	14.3	−2.1**	1.2	0.07
$35,000–$49,999	12.4	11.0	−1.4**	11.3	10.1	−1.2**	−0.2	0.89
$50,000–$74,999	14.3	13.9	−0.4	12.1	12.5	0.4	−0.8	0.30
≥$75,000	25.6	32.5	6.9**	29.3	33.1	3.8**	3.1	0.001
Missing	13.6	16.8	3.2**	14.6	19.6	5.0**	−1.8	0.06

* Provided regular care or assistance during the previous 30 days to a friend or family member who had a health problem or disability.

^†^ Alabama, Arizona, Arkansas, California, Colorado, Connecticut, Georgia, Hawaii, Idaho, Illinois, Indiana, Iowa, Kentucky, Louisiana, Maine, Maryland, Minnesota, Mississippi, Missouri, Nebraska, Nevada, New Jersey, New York, North Dakota, Ohio, Oregon, Pennsylvania, South Carolina, South Dakota, Texas, Utah, Virginia, West Virginia, Wisconsin, and Wyoming.

^§^ Percentages are weighted and unadjusted.

^¶^ Using logistic regression model with caregiving status (dependent variable), demographic indicator (independent variable), time period indicator (independent variable), and an interaction term for demographic × time.

** p<0.05, using *t*-test with Taylor series linearization.

^††^ Adults of Hispanic or Latino (Hispanic) origin might be of any race but are categorized as Hispanic; all racial groups are non-Hispanic.

Four health indicators improved among caregivers from 2015–2016 to 2021–2022: decrease in prevalence of current smoking, physical inactivity, no health coverage, and inability to see a doctor due to cost. Six indicators worsened: the prevalences of frequent mental distress, depression, asthma, obesity, and having any or multiple chronic physical conditions all increased (Table 2).

**TABLE 2:** Changes in prevalence of selected health indicators among caregivers* and noncaregivers — Behavioral Risk Factor Surveillance System, 35 states^†^ and Puerto Rico, 2015–2016 and 2021–2022

Health indicator	% (95% CI)^§^		Change from 2015–2016 to 2021–2022		Difference in change (caregivers minus noncaregivers)	
	2015–2016	2021–2022	Percentage point change, unadjusted	Age-adjusted p-value^¶^	Percentage point	Age-adjusted p-value**
**Behavior**						
**Current cigarette smoking**						
Caregivers	20.9**^††^** (19.7–22.0)	16.6**^††^** (15.8–17.4)	−4.3**^§§^**	<0.001	−1.0	0.70
Noncaregivers	15.0 (14.5–15.5)	11.7 (11.4–12.1)	−3.3**^§§^**	<0.001		
**Binge drinking^¶¶^**						
Caregivers	15.3 (14.1–16.4)	14.2 (13.4–15.1)	−1.1	0.87	0.4	0.11
Noncaregivers	16.6 (16.1–17.1)	15.1 (14.6–15.5)	−1.5**^§§^**	<0.001		
Heavy drinking***						
Caregivers	5.9 (5.2–6.7)	6.5**^††^**(5.7–7.2)	0.6	0.13	0.6	0.23
Noncaregivers	5.7(5.4–6.1)	5.7(5.4–6.0)	0	0.95		
**Physical inactivity^†††^**						
Caregivers	24.0**^††^** (22.8–25.2)	22.0**^††^** (21.1–22.9)	−2.0**^§§^**	<0.001	−1.2	0.03
Noncaregivers	25.8 (25.2–26.4)	25.0 (24.5–25.6)	−0.8	0.02		
**General/Mental health**						
**Fair or poor self-rated health**						
Caregivers	19.7**^††^** (18.6–20.7)	19.6**^††^** (18.7–20.4)	−0.1	0.34	0.5	0.81
Noncaregivers	17.5 (17.0–18.0)	16.9 (16.4–17.4)	−0.6	0.01		
**Frequent mental distress^§§§^**						
Caregivers	17.2**^††^** *(*16.1–18.3)	20.5**^††^** (19.5–21.5)	3.3**^§§^**	<0.001	−0.3	0.15
Noncaregivers	10.0 (9.6–10.5)	13.6 (13.1–14.0)	3.6**^§§^**	<0.001		
**Frequent physical distress^¶¶¶^**						
Caregivers	14.7**^††^** (13.7–15.7)	14.3**^††^** (13.5–15.0)	−0.4	0.17	0.2	0.71
Noncaregivers	11.8 (11.3–12.2)	11.2 (10.8–11.5)	−0.6**^§§^**	0.005		
Depression****						
Caregivers	23.3**^††^** (22.2–24.4)	25.6**^††^** (24.6–26.6)	2.3**^§§^**	<0.001	−1.5	0.007
Noncaregivers	14.8 (14.4–15.3)	18.6 (18.1–19.0)	3.8**^§§^**	<0.001		
**Chronic physical conditions^††††^**						
**CHD**						
Caregivers	7.3**^††^** (6.7–7.8)	7.3 (6.8–7.8)	0	0.02	0.1	0.17
Noncaregivers	6.4 (6.1–6.7)	6.3 (6.1–6.6)	−0.1	0.04		
**Stroke**						
Caregivers	3.5 (3.0–3.9)	3.8 (3.3–4.2)	0.3	0.84	0.1	0.51
Noncaregivers	3.1 (2.9–3.3)	3.3 (3.1–3.5)	0.2	0.99		
**COPD**						
Caregivers	8.2**^††^** (7.6–8.9)	9.1**^††^** (8.5–9.7)	0.9**^§§^**	0.49	0.7	0.83
Noncaregivers	6.0 (5.7–6.3)	6.2 (5.9–6.4)	0.2	0.80		
**Arthritis**						
Caregivers	32.6**^††^** (31.3–33.9)	34.8**^††^** (33.7–35.9)	2.2**^§§^**	0.36	1.5	0.28
Noncaregivers	23.8 (23.4–24.3)	24.5 (24.0–25.0)	0.7	0.52		
**Diabetes**						
Caregivers	11.2 (10.5–11.9)	12.9 (12.2–13.5)	1.7**^§§^**	0.36	1.0	0.95
Noncaregivers	11.2 (10.8–11.6)	11.9 (11.5–12.3)	0.7**^§§^**	0.27		
**Current asthma**						
Caregivers	11.6**^††^** (10.7–12.4)	12.8**^††^** (12.0–13.5)	1.2**^§§^**	0.009	0.4	0.70
Noncaregivers	8.3 (8.0–8.7)	9.1 (8.7–9.4)	0.8**^§§^**	0.005		
**Obesity**						
Caregivers	34.1**^††^** (32.7–35.4)	38.0**^††^** (36.8–39.2)	3.9**^§§^**	<0.001	0.1	0.80
Noncaregivers	29.4 (28.8–30.1)	33.2 (32.6–33.8)	3.8**^§§^**	<0.001		
**Any chronic physical condition**						
Caregivers	60.6**^††^** (59.2–62.1)	65.7**^††^** (64.4–66.9)	5.1**^§§^**	<0.001	1.9	0.79
Noncaregivers	51.7 (51.0–52.5)	54.9 (54.3–55.5)	3.2**^§§^**	<0.001		
**Multiple chronic physical conditions**						
Caregivers	28.9**^††^** (27.7–30.1)	32.5**^††^** (31.3–33.6)	3.6**^§§^**	0.04	1.4	0.40
Noncaregivers	22.0 (21.5–22.5)	24.2 (23.6–24.7)	2.2**^§§^**	<0.001		
**Health care access**						
**No health coverage (age <65 yrs)^§§§§^**						
Caregivers	14.1 (12.8–15.4)	9.0^††^ (8.2–9.8)	−5.1**^§§^**	<0.001	−2.5	0.006
Noncaregivers	13.7 (13.0–14.3)	11.1 (10.6–11.6)	−2.6**^§§^**	<0.001		
**Inability to see doctor due to cost^¶¶¶¶^**						
Caregivers	16.6^††^ (15.6–17.7)	13.2^††^ (12.4–14.0)	−3.4**^§§^**	<0.001	−0.8	0.36
Noncaregivers	11.7 (11.3–12.2)	9.1 (8.8–9.5)	−2.6**^§§^**	<0.001		

**Abbreviations:** CHD = coronary heart disease; COPD = chronic obstructive pulmonary disease; HMO = health management organization.

* Provided regular care or assistance during the previous 30 days to a friend or family member who had a health problem or disability.

^†^ Alabama, Arizona, Arkansas, California, Colorado, Connecticut, Georgia, Hawaii, Idaho, Illinois, Indiana, Iowa, Kentucky, Louisiana, Maine, Maryland, Minnesota, Mississippi, Missouri, Nebraska, Nevada, New Jersey, New York, North Dakota, Ohio, Oregon, Pennsylvania, South Carolina, South Dakota, Texas, Utah, Virginia, West Virginia, Wisconsin, and Wyoming.

^§^ Percentages are weighted and unadjusted; CIs account for complex sample design.

^¶^ Using logistic regression model with health indicator (dependent variable), period indicator (independent variable), and continuous age (independent variable). Significant results indicate the percentage during 2015–2016 was different compared with 2021–2022 after age adjustment (p<0.05).

** Age adjusted time interaction, using logistic regression model with health indicator (dependent variable), caregiving status (independent variable), time period indicator (independent variable), continuous age (independent variable), and an interaction term for caregiving × time.

**^††^** p<0.05, using logistic regression model with health indicator (dependent variable), caregiving status (independent variable), and continuous age (independent variable). Significant results indicate the percentage for caregivers was different from noncaregivers after age adjustment.

^§§^ Statistically significant difference from 2015–2016 to 2021–2022 using *t*-test with Taylor series linearization.

^¶¶^ Five or more drinks on at least one occasion for men or four or more drinks for women during the previous 30 days.

*** Fifteen or more drinks per week for men; eight or more drinks per week for women during the previous 30 days.

^†††^ No leisure-time physical activity during the previous 30 days.

^§§§^ A response of ≥14 days to the question, “Now thinking about your mental health, which includes stress, depression, and problems with emotions, for how many days during the past 30 days was your mental health not good?”

^¶¶¶^ A response of ≥14 days to the question, “Now thinking about your physical health, which includes physical illness and injury, for how many days during the past 30 days was your physical health not good?”

**** Respondents self-reported diagnosis of depression in their lifetime.

^††††^ Respondents reported diagnosis of physical chronic conditions in their lifetime for CHD (including myocardial infarction or angina), stroke, COPD, arthritis (including rheumatoid arthritis, gout, lupus, or fibromyalgia), or diabetes (excluding those who reported only gestational diabetes, prediabetes, or borderline diabetes) and currently having asthma. Obesity was defined as having a body mass index ≥30.0 kg/m^2^ based on self-reported height and weight.

^§§§§^ During 2015–2016, responded no to “Do you have any kind of health care coverage, including health insurance, prepaid plans such as HMOs, government plans such as Medicare, or Indian Health Service?” During 2021–2022, responded “no coverage of any type” to “What is the current primary source of your health insurance?”

^¶¶¶¶^ During 2015–2016, the question was “Was there a time in the past 12 months when you needed to see a doctor but could not because of cost?” During 2021–2022, the end of the question was revised to “… because you could not afford it?”

From 2015–2016 to 2021–2022, the prevalence of current smoking decreased among both caregivers and noncaregivers (Table 2). Caregivers were more likely than were noncaregivers to smoke during both periods. The prevalence of physical inactivity decreased for both caregivers and noncaregivers; the decrease for caregivers was larger (2.0 versus 0.8 percentage points; age-adjusted time interaction p = 0.03).

From 2015–2016 to 2021–2022, the prevalence of frequent mental distress increased among both caregivers and noncaregivers (Table 2). The prevalence of lifetime diagnosed depression increased from 2015–2016 to 2021–2022 for both noncaregivers (3.8 percentage points) and caregivers (2.3 percentage points; age-adjusted time interaction p = 0.007). Both mental health indicators (frequent mental distress and depression) were more prevalent among caregivers when compared with noncaregivers during both periods.

During 2021–2022, measures for 13 of the 19 health indicators were unfavorable for caregivers when compared with noncaregivers. Four of the seven chronic physical conditions were more common among caregivers in both periods: obesity, current asthma, chronic obstructive pulmonary disease, and arthritis. The prevalence of obesity and current asthma increased for both caregivers and noncaregivers; the prevalence of chronic obstructive pulmonary disease and arthritis did not change.

The percentage of adults aged <65 years with no health care coverage was similar among caregivers and noncaregivers during 2015–2016 and decreased during 2021–2022 among both groups (caregivers 5.1 percentage points and noncaregivers 2.6 percentage points; age-adjusted time interaction p = 0.006) (Table 2). Caregivers were more likely than noncaregivers to report inability to see a doctor due to cost during both periods.

## Discussion

A positive social connection can grow between a caregiver and care recipient, providing a sense of purpose for the caregiver and less stress for the care recipient (*4*). However, caregivers had worse age-adjusted outcomes for 13 of the 19 health indicators examined during 2021–2022. Many of these findings are consistent with previous reports, including the association between caregiving and smoking (*5*,*6*), poor mental health (*3*,*4*,*7*), obesity (*5*), and asthma (*3*).

Overarching circumstances during the time of the study, including the COVID-19 pandemic and health care reform policies, affected the U.S. population. Increased prevalence of lifetime depression and frequent mental distress among both caregivers and noncaregivers is consistent with findings of population-level mental health impacts during the COVID-19 pandemic (*8*). Expanded eligibility for Medicaid occurred during the study period,**** and national data indicate that public health plan coverage (including Medicaid) increased among adults aged <65 years (*9*).

The National Strategy to Support Family Caregivers has raised awareness of the need to support the health of caregivers nationwide. Goals outlined in the strategy include strengthening services and supports for family caregivers and expanding data, research, and evidence-based practices (*2*). Providing relief from caregiving tasks, broadly known as “respite care,” was identified as a priority. Availability of such services can be optimized through public policies and community collaboration, resulting in high-quality, affordable, and flexible care (*2*). Additional strategies to ensure financial and workplace security for caregivers have been implemented in some states, and include enhancement of paid family leave and antidiscrimination laws.^††††^

### Limitations

The findings in this report are subject to at least four limitations. First, all measures are self-reported and might be subject to recall and social desirability bias, possibly resulting in misclassification of health indicators. Second, data were not available in all states, and estimates might not be representative of all U.S. adults. Third, the study design was cross-sectional at two time points rather than longitudinal (i.e., survey participants were not followed over time to observe changes). Finally, this study was descriptive and further adjustment might explain differences between caregivers and noncaregivers. Despite these limitations, this study is the first to examine changes in the health of caregivers from a population health perspective using a large sample.

### Implications for Public Health Practice

National, state, and local public health strategies that address comprehensive chronic disease prevention and management^§§§§^ could be tailored for caregivers. Many community organizations support caregivers by offering interventions designed to relieve caregiver strain, including skills training, support groups, and care coordination.^¶¶¶¶^ Specialized training designed to help caregivers cope with the unique challenges of dementia care can be especially helpful for persons who care for those with memory loss or cognitive decline.***** In health care settings, professionals can take steps to identify patients who serve in a caregiving role and encourage them to seek any support they might need to prioritize their own mental and physical health. Additional critical strategies outlined in the National Strategy to Support Family Caregivers call on public and private sectors to provide resources for caregivers (*2*).

## Footnotes

*
https://www.cdc.gov/brfss/

† States included Alabama, Arizona, Arkansas, California, Colorado, Connecticut, Georgia, Hawaii, Idaho, Illinois, Indiana, Iowa, Kentucky, Louisiana, Maine, Maryland, Minnesota, Mississippi, Missouri, Nebraska, Nevada, New Jersey, New York, North Dakota, Ohio, Oregon, Pennsylvania, South Carolina, South Dakota, Texas, Utah, Virginia, West Virginia, Wisconsin, and Wyoming. If a state or territory completed both years within a single period (e.g., 2015 and 2016), only the most recent year was retained.

§ 45 C.F.R. part 46.102(l)(2), 21 C.F.R. part 56; 42 U.S.C. Sect. 241(d); 5 U.S.C. Sect. 552a; 44 U.S.C. Sect. 3501 et seq.

¶ Five or more drinks on at least one occasion for men or four or more drinks for women during the previous 30 days.

** Fifteen or more drinks per week for men; eight or more drinks per week for women during the previous 30 days.

†† No leisure-time physical activity during the previous 30 days.

§§ A response of ≥14 days to the question, “Now thinking about your mental health, which includes stress, depression, and problems with emotions, for how many days during the past 30 days was your mental health not good?”

¶¶ A response of ≥14 days to the question, “Now thinking about your physical health, which includes physical illness and injury, for how many days during the past 30 days was your physical health not good?”

*** Respondents self-reported diagnosis of depression in their lifetime.

††† Respondents reported diagnosis of physical chronic conditions in their lifetime for coronary heart disease (including myocardial infarction or angina), stroke, chronic obstructive pulmonary disease, arthritis (including rheumatoid arthritis, gout, lupus, or fibromyalgia), or diabetes (excluding those who reported only gestational diabetes, prediabetes, or borderline diabetes) and currently having asthma. Obesity was defined as having a body mass index ≥30.0 kg/m^2^ based on self-reported height and weight.

§§§ During 2015–2016, responded no to the question, “Do you have any kind of health care coverage, including health insurance, prepaid plans such as HMOs, government plans such as Medicare, or Indian Health Service?” During 2021–2022, responded “no coverage of any type” to the question, “What is the current primary source of your health insurance?”

¶¶¶ During 2015–2016, the question was “Was there a time in the past 12 months when you needed to see a doctor but could not because of cost?” During 2021–2022, the end of the question was revised to “… because you could not afford it?”

****
https://www.kff.org/affordable-care-act/issue-brief/status-of-state-medicaid-expansion-decisions-interactive-map/

††††
https://nashp.org/financial-and-workplace-security-for-family-caregivers/

§§§§ https://www.cdc.gov/nccdphp/impact/index.html; https://www.astho.org/topic/population-health-prevention/chronic-disease/

¶¶¶¶
https://www.caregiver.org/connecting-caregivers/

*****
https://bpc.caregiver.org/pro/index.html#searchPrograms
